# Effects of Intercropping and Mowing Frequency on Biological Nitrogen Fixation Capacity, Nutritive Value, and Yield in Alfalfa (*Medicago sativa* L. cv. Vernal)

**DOI:** 10.3390/plants14020240

**Published:** 2025-01-16

**Authors:** Yao Wang, Jinsong Zhang, Chunxia He, Ping Meng, Jie Wang, Jun Gao, Pan Xue

**Affiliations:** 1 Key Laboratory of Tree Breeding and Cultivation, National Forestry and Grassland Administration, Research Institute of Forestry, Chinese Academy of Forestry, Beijing 100091, China; wy96157@163.com (Y.W.); zhangjs@caf.ac.cn (J.Z.); mengping@caf.ac.cn (P.M.); gaojun@caf.ac.cn (J.G.); 2 Collaborative Innovation Center of Sustainable Forestry in Southern China, Nanjing Forest University, Nanjing 210037, China; 3 Henan Xiaolangdi Forest Ecosystem National Observation and Research Station, Jiyuan 454650, China; 4 Academy of Forestry Inventory and Planning, State Forestry and Grassland Administration, Beijing 100714, China; wj18800128612@126.com; 5 Jiyuan Forestry Bureau, Jiyuan 454650, China; jonathanknow@126.com

**Keywords:** intercropping, mowing frequency, biological nitrogen fixation capacity, yield, nutritive value

## Abstract

Intercropping with legume forages is recognized as an effective strategy for enhancing nitrogen levels in agroforestry, while mowing may influence nitrogen fixation capacity and yield. This study investigated the rooting, nitrogen fixation, nutritive value, and yield of alfalfa (*Medicago sativa* L.) under intercropping and varying mowing frequencies (CK, 2, and 3) from 2021 to 2023, using walnut (*Juglans regia* L.) and alfalfa as experimental subjects. The results indicated that intercropping suppressed root growth, whereas increased mowing frequency stimulated root development in the topsoil (0–20 cm). Specifically, the average root length density, root surface area, and root volume from the twice- and thrice-mowed treatments increased by 18.26, 17.45, and 4.15%, respectively, in comparison to the control. The δ^15^N values of the intercropped alfalfa were significantly lower than those of the monocropped alfalfa (*p* < 0.05), with the δ^15^N values of the mowing-thrice treatment increasing by an average of 38.61% compared to the control. Intercropping suppressed alfalfa yield but did not affect the total nitrogen content in the leaves or the nutritive value, and all mowing treatments resulted in land equivalent ratios (LERs) greater than 1. Furthermore, increased mowing frequency enhanced both the nutritive value and yield of alfalfa. Our study suggests that intercropping with walnut can improve biological nitrogen fixation in alfalfa, and that adopting a mowing-thrice regime can optimize yield and nutritive value.

## 1. Introduction

Walnut (*Juglans regia* L.) trees are important economic species characterized by their robust, durable wood and nutritious fruits, and they are cultivated worldwide [1]. However, walnut plantations are usually established as monocultures, leading to issues in the understory such as bare soil and weed competition, which lead to fragile ecosystems and degraded soil quality [2,3]. To address these challenges, the effective management of understory planting is essential. Currently, understory management employs various methods, including manual removal, chemical herbicide application, and agroforestry practices, with agroforestry demonstrating distinct advantages over the other approaches [4]. Agroforestry refers to the integration of perennial trees with crops or herbaceous plants within the same area [5]. Numerous studies have demonstrated that agroforestry systems can significantly enhance land use efficiency and optimize resource allocation [6,7]. Therefore, selecting appropriate intercropping plants within walnut plantations is a vital strategy for understory management, which can stabilize ecological structures and enhance economic benefits.

Legumes are valued for their ability to fix atmospheric nitrogen, making them a critical consideration when selecting plant material for intercropping. Over the long term, legumes significantly contribute nitrogen to the ecosystem, which non-nitrogen-fixing plants can subsequently absorb and utilize through the decomposition of legume residues and root exudates [8,9]. As the cost of chemical nitrogen fertilizers continues to rise and environmental pollution issues worsen, the significance of legumes in future tree–crop intercropping systems is likely to increase [10]. Alfalfa (*Medicago sativa* L.), a perennial leguminous herb, offers several advantages, including a high capacity for biological nitrogen fixation, substantial forage yields, and rich nutritive value [11]. It is an appropriate choice for intercropping with walnuts and has been widely implemented in various agroforestry practices [12,13].

Mutual benefits and competition coexist among plants within ecosystems, and the effectiveness of intercropping systems hinges on maximizing mutual benefits while minimizing competition [14]. In the intercropping system involving walnut and alfalfa, nitrogen—particularly effective nitrogen fixation—constitutes a critical area of research [15]. Fully utilizing the biological nitrogen fixation capacity of alfalfa is essential for enhancing the nutrient dynamics of ecosystems and fostering interspecies mutual-benefit relationships. However, controversies persist, and limited studies have examined how intercropping impacts the nitrogen-fixing capacity of legumes. Some studies suggest that competition with trees may inhibit root development and root nodule activity in legumes [16], while others indicate that intercropping with walnut can enhance the biological nitrogen fixation capacity of alfalfa [17]. Additionally, the formation of legume root nodules necessitates specific conditions and time. McKenzie et al. [18] found that the natural formation of root nodules is more challenging when legumes are cultivated in previously unplanted soil, and different rhizobia species exert varying effects on nodulation effectiveness [19]. The allelopathic effects of walnuts should not be overlooked, as they may inhibit root growth and nodulation in alfalfa [17]. Consequently, it cannot be assumed that the inclusion of alfalfa will positively influence nitrogen accumulation in ecosystems. Accurate quantitative studies on biological nitrogen fixation by alfalfa in intercropping systems, utilizing the δ^15^N natural abundance method or other technological means, are essential for understanding the interspecific nitrogen relationships in these systems. The δ^15^N method has been widely used in recent years in studies related to nitrogen fixation, and δ^15^N values can be used as an indicator to characterize nitrogen fixation capacity [17,20].

Mowing is a common management practice in alfalfa cultivation, and it is widely accepted that mowing positively influences forage yield and quality development. In practice, increased mowing frequency is often employed to achieve greater economic benefits [21]. The study by Li et al. [22] demonstrated that multiple cuttings increased alfalfa yields by 82–114% while reducing acid and neutral detergent fiber content compared to a single harvest. Similarly, research on *Panicum antidotale* Retz revealed that mowing every 40 days maximized forage yield and protein content [23]. However, the effects of mowing on alfalfa are not universally positive. Excessive mowing frequency or intensity may lead to root nutrient deficiencies, adversely affecting rhizome formation as well as forage yield and quality [24]. Moreover, the increased biomass of alfalfa resulting from mowing can lead to higher water consumption, potentially exacerbating resource competition within intercropping systems [25].

In light of this background, the present study aimed to understand the effects of intercropping and mowing frequency on the nitrogen fixation, yield, and quality of alfalfa. The primary objectives of this study were the following: (1) to determine whether intercropping and mowing could enhance the nitrogen fixation capacity of alfalfa; (2) to investigate the impact of different mowing frequencies on alfalfa’s nutritive value and yield; and (3) to evaluate the land use efficiency of intercropping versus monocropping systems at various mowing frequencies.

## 2. Results

### 2.1. Morphological Characteristics of Alfalfa Root System

The vertical distribution characteristics of the root systems of monocropped and intercropped alfalfa were similar, exhibiting the highest root length density, root surface area, average root diameter, and root volume in the 0–20 cm soil layer (Table 1). The above-root morphological indexes decreased progressively with increasing soil depth, with significant differences (*p* < 0.05) observed among different soil layers. Compared to the 0–20 cm soil layer, the mean root length density, root surface area, average root diameter, and root volume in the 60–80 cm soil layer decreased by 84.96, 84.99, 76.14, and 84.58%, respectively. Furthermore, the root length density, root surface area, average root diameter, and root volume of monocropped alfalfa were significantly higher (*p* < 0.05) than those of intercropped alfalfa in all soil layers (Table 1).

In different soil layers, the response of alfalfa root morphology varied with mowing frequency. In the 0–20 cm soil layer, compared to the CK treatment, mowing twice and thrice had no obvious influence on the average root diameter, but significantly increased root length density (by 17.39 and 19.13% for MA-2 and MA-3, and 27.71 and 25.30% for IA-2 and IA-3, respectively), root surface area (by 15.33 and 19.56% for MA-2 and MA-3, and 14.54 and 11.99% for IA-2 and IA-3, respectively), and root volume (by 3.80 and 4.50% for MA-2 and MA-3, and 4.33 and 3.98% for IA-2 and IA-3, respectively). In the 20–40 cm soil layer, increased mowing frequency significantly reduced average root diameter (17.65 and 28.96% for MA-2 and MA-3, respectively, compared to MA-CK; and 11.05 and 23.26% for IA-2 and IA-3, respectively, compared to IA-CK), while root length density, root surface area, and root volume were unaffected. In both the 40–60 and 60–80 cm soil layers, the root length density, root surface area, average root diameter, and root volume of both the monocropped and intercropped alfalfa significantly decreased with increasing mowing frequency. This finding indicates that increased mowing frequency generally inhibited root growth in alfalfa; meanwhile, it had a positive effect on the root system in the surface soil layer, resulting in an upward shift in the overall distribution of the root system.

### 2.2. Changes in δ^15^N Values in Alfalfa Stems and Leaves

**Figure 1:**
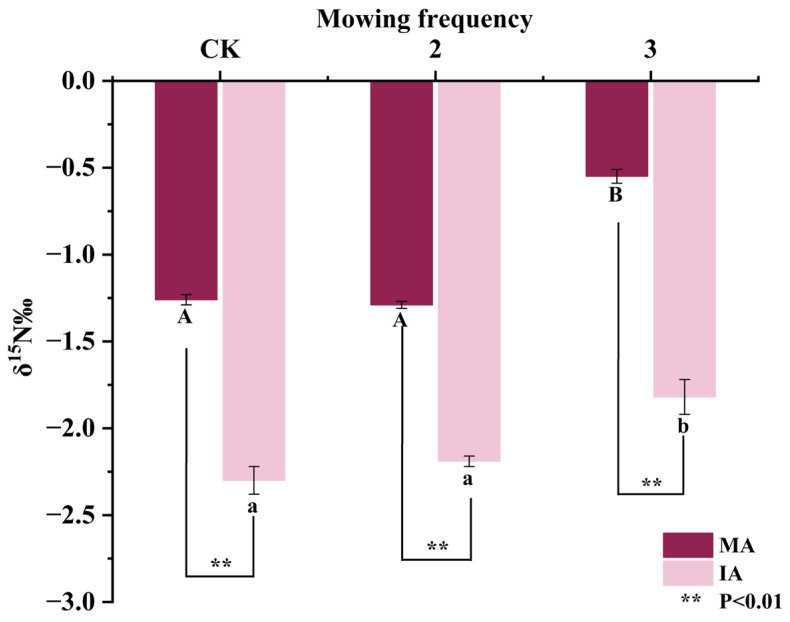
Changes in δ^15^N values of alfalfa under different mowing frequencies. Different lowercase letters indicate significant differences (*p* < 0.05), and error bars represent standard deviation. CK, 2, and 3 indicate mowing-once, -twice, and -thrice treatments, respectively; MA and IA indicate monocropped and intercropped alfalfa.

The mean δ^15^N value of *Festuca elata* was 1.73 ± 0.20‰ (Supplementary Table S2), whereas the mean δ^15^N values of both the monocropped and intercropped alfalfa were less than 0 (−1.03‰ and −2.10‰, respectively), which demonstrated the effectiveness of biological N fixation in alfalfa. The δ^15^N signatures of the monocropped and intercropped alfalfa responded consistently to mowing frequency, both exhibiting significantly higher (*p* < 0.05) ^15^N values in the mowed-thrice treatments than in the CK and mowed-twice treatments (nonsignificant difference). Compared to CK, the δ^15^N values of MA-3 and IA-3 increased by 56.35 and 20.87%, respectively (Figure 1). Among all treatments, the δ^15^N values of the intercropped alfalfa were highly significantly lower than the δ^15^N values of the monocropped alfalfa (*p* < 0.01). Compared to the monocropped alfalfa, the δ^15^N values of the intercropped alfalfa were reduced by 82.54, 69.77, and 230.91% in the CK, mowing-twice, and mowing-thrice treatments, respectively, which suggests that intercropping with walnut improves the biological nitrogen fixation capacity of alfalfa.

### 2.3. Changes in Total Nitrogen Content of Leaves in Alfalfa

**Figure 2:**
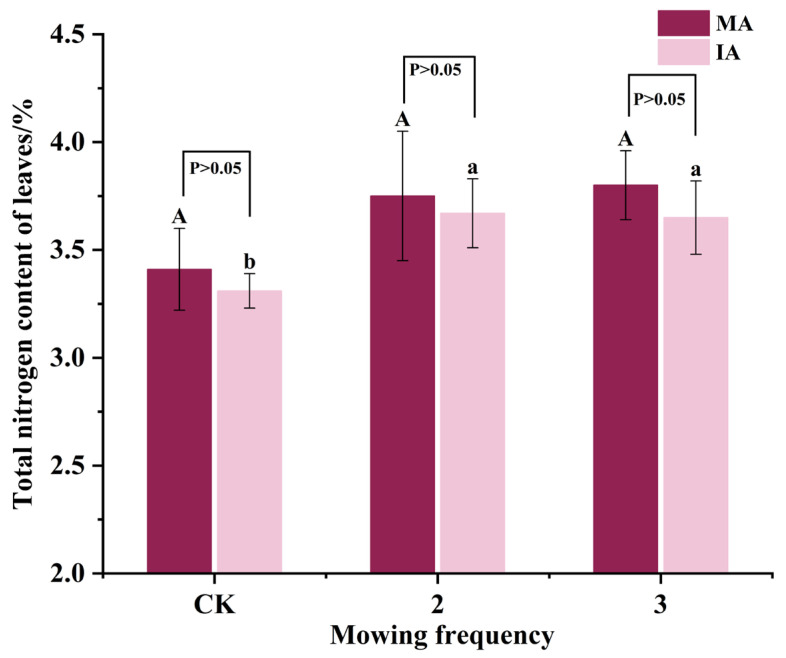
Changes in total nitrogen contents of leaves in alfalfa. Different lowercase letters indicate significant differences (*p* < 0.05), and error bars represent standard deviation. CK, 2, and 3 indicate mowing-once, -twice and -thrice treatments, respectively; MA and IA indicate monocropped and intercropped alfalfa.

The total nitrogen contents of the leaves in both the mowing-twice and -thrice treatments were significantly higher than those of CK (*p* < 0.05), and there was no difference between the mowing-twice and -thrice treatments (*p* > 0.05). For the monocropped alfalfa, MA-2 and MA-3’s values were 9.97 and 11.44% higher compared to MA-CK, respectively (Figure 2); for the intercropped alfalfa, IA-2 and IA-3’s values were 10.88 and 10.27% higher compared to IA-CK, respectively. There was no significant difference (*p* > 0.05) in leaf total nitrogen between the monocropped and intercropped alfalfa in all treatments.

### 2.4. Nutritive Value and Stem-to-Leaf Ratio of Alfalfa

**Figure 3:**
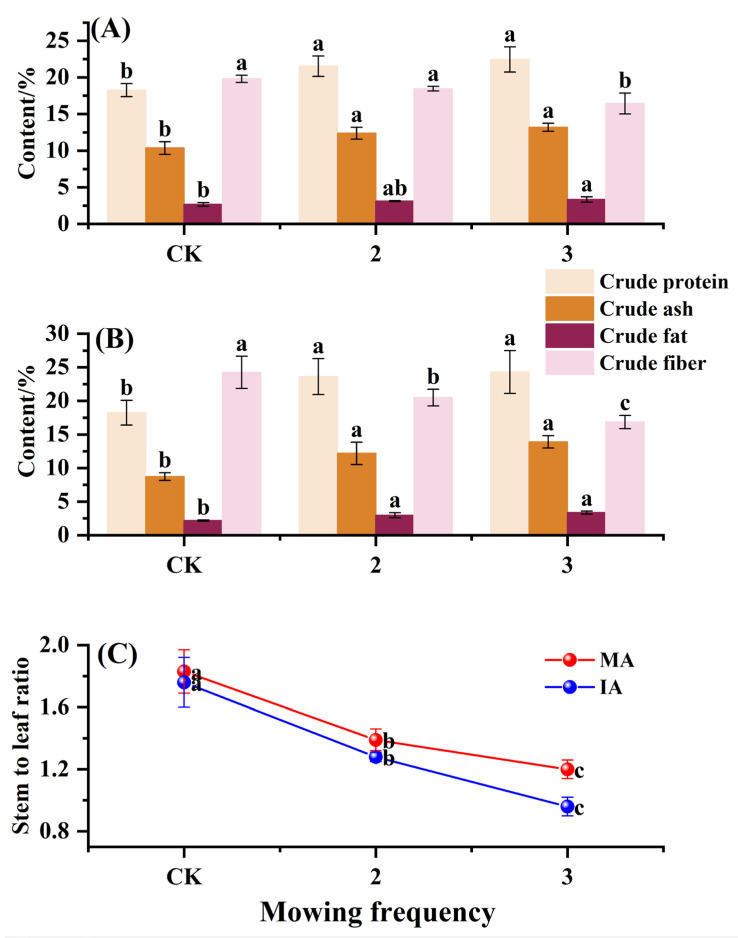
Nutritive value and stem-to-leaf ratio of alfalfa at different mowing frequencies: crude protein, crude ash, crude fat, and crude fiber contents of alfalfa at different mowing frequencies in (**A**) monocropping system and (**B**) intercropping system. (**C**) Stem-to-leaf ratio at different mowing frequencies. Different lowercase letters indicate significant differences (*p* < 0.05), and error bars represent standard deviation. CK, 2, and 3 indicate mowing-once, -twice, and -thrice treatments, respectively; MA and IA indicate monocropped and intercropped alfalfa.

For the monocropped alfalfa, the CK treatment had the lowest crude protein and crude ash contents, which were 17.91% and 22.89% less than those in the mowing twice treatment, 19.59% and 27.32% less than those in the mowing thrice treatment respectively, and there were no significant differences between the mowing-twice and -thrice treatments (Figure 3A); the crude fat content of the mowing-thrice treatment was higher than that of CK (increased by 24.72%, *p* < 0.05), whereas the differences were not significant in the mowing-twice compared to both the CK and mowing-thrice treatments; the crude fiber content was the lowest in the mowing thrice treatment, and its content in the CK and mowing-twice treatments had no significant difference.

For intercropped alfalfa, the crude protein, crude ash and crude fat contents showed similar responses to mowing frequency, as evidenced by the fact that the mowing-twice and -thrice treatments were significantly higher than CK (*p* < 0.05), and the former two treatments had no significant difference (*p* > 0.05); the crude fiber content continued to decrease with the increase in mowing frequency, and the differences were significant among all treatments (*p* < 0.05), with the values in the mowing-twice and -thrice treatments decreasing by 15.51 and 30.52% compared to CK, respectively (Figure 3B). In addition, the differences in the mean crude protein, crude ash, crude fat, and crude fiber contents between the monocropped and intercropped alfalfa were not significant (*p* > 0.05).

The stem-to-leaf ratio of both the monocropped and intercropped alfalfa continued to decrease with increasing frequencies of mowing, and the difference was significant among all treatments (*p* < 0.05). There was a 24.04 and 34.43% decrease in the stem-to-leaf ratio in the MA-2 and MA-3 treatments, respectively (Figure 3C), and a 27.27 and 45.45% decrease in that in the IA-2 and IA-3 treatments, respectively, compared to MA-CK.

### 2.5. Annual Average Yield Changes in Walnut and Alfalfa and Calculation of LER

**Figure 4:**
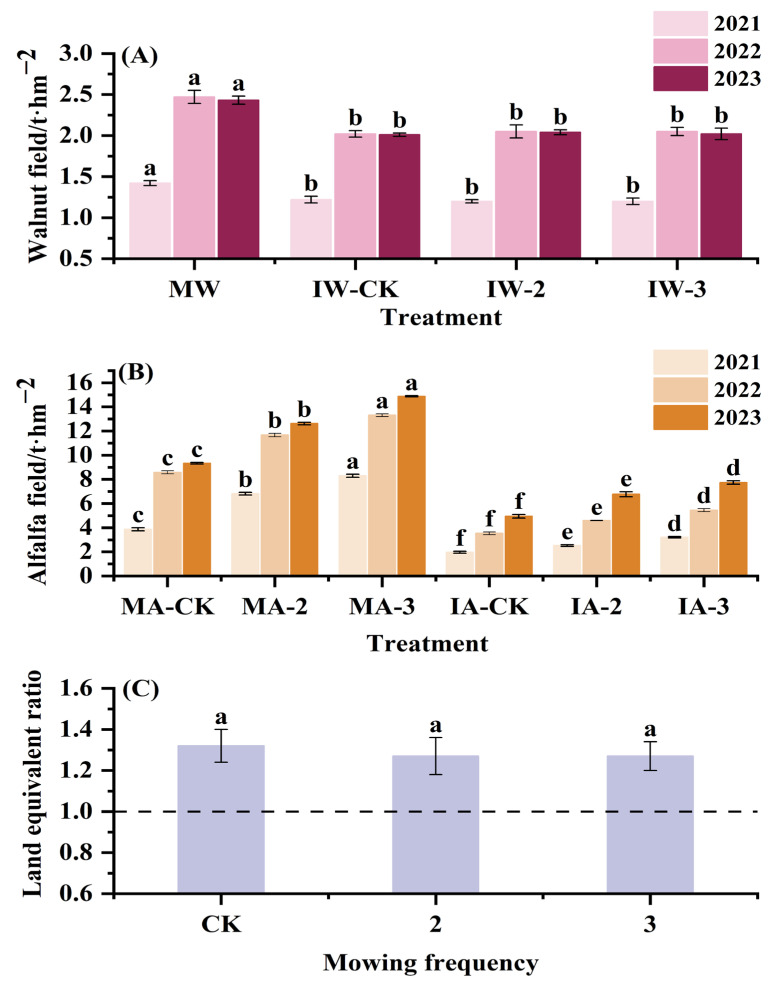
Annual average yield variation in walnut and alfalfa and calculation of land equivalent ratio: (**A**) yield of monocropped and intercropped walnut at different mowing frequencies; (**B**) yield of monocropped and intercropped alfalfa at different mowing frequencies; (**C**) LER at different mowing frequencies. Different lowercase letters indicate significant differences (*p* < 0.05), and error bars represent standard deviation. MW, IW-CK, IW-2, and IW-3 indicate monocropped walnut and intercropped walnut subjected to mowing once, twice, and thrice, respectively; MA-CK, MA-2, and MA-3 represent monocropped alfalfa subjected to mowing once, twice, and thrice, respectively; IA-CK, IA-2, and IA-3 represent intercropped alfalfa subjected to mowing once, twice, and thrice, respectively; and CK, 2, and 3 indicate mowing-once, -twice and -thrice treatments, respectively.

The comparison results of the walnut and alfalfa yields from 2021 to 2023 showed that the monocropped walnut yield was significantly higher than the intercropped walnut yield (*p* < 0.05), mowing frequency did not have a significant effect on walnut yield (the difference between the walnut yields of IW-CK, IW-2, and IW-3 was not significant) (*p* > 0.05), and walnut yield was lower in 2021 compared to 2022 and 2023 (Figure 4A). The yields of both the monocropped and intercropped alfalfa increased with increasing mowing frequency, and the difference among all treatments was significant (*p* < 0.01). Alfalfa yield increased by 42.84 and 67.49% in the MA-2 and MA-3 treatments, respectively, compared to MA-CK, as well as by 33.05 and 57.18% in the IA-2 and IA-3 treatments, respectively, compared to IA-CK (Figure 4B). In addition, alfalfa yield was significantly higher (*p* < 0.05) in both of the monocropped fields than in the intercropped one at the same mowing frequency. The results of the annual average land equivalent ratio (LER) of different treatments showed that the LER of the CK (1.32), mowed-twice (1.27) and mowed-thrice (1.27) treatments were all greater than 1 (Figure 4C), and were not significantly influenced by mowing frequency (*p* > 0.05).

### 2.6. Correlation Analysis Among Alfalfa Yield, δ^15^N, and Other Indexes

**Figure 5:**
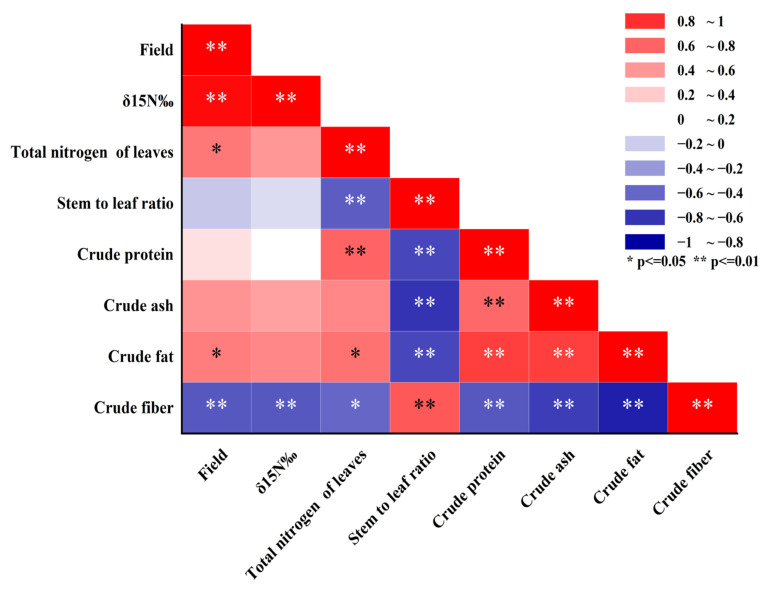
Correlation analysis between eight indicators, including yield. *: *p* < 0.05 and **: *p* < 0.01.

The results of the correlation analysis showed a complex correlation among the yield, δ^15^N, and nutritive value of alfalfa (Figure 5). Yield was significantly positively correlated with δ^15^N (r = 0.77, *p* < 0.01), significantly positively correlated with the total nitrogen of the leaves and the crude fat (r = 0.55 and 0.48, *p* < 0.05), and strongly significantly negatively correlated with crude fiber (r = −0.66, *p* < 0.01). δ^15^N was strongly significantly negatively correlated with crude fiber (r = −0.65, *p* < 0.01). The total nitrogen of the leaves was strongly significantly positively correlated with crude protein (r = 0.6, *p* < 0.01), significantly positively correlated with crude fat (r = 0.55, *p* < 0.05), strongly significantly negatively correlated with the stem-to-leaf ratio (r = −0.62, *p* < 0.01), and significantly negatively correlated with crude fiber (r = −0.58, *p* < 0.05). The correlations between nutritive indexes, such as the stem-to-leaf ratio and crude protein, were strongly significant (*p* < 0.01): the stem-to-leaf ratio and crude fiber were positively correlated; they were negatively correlated with crude protein, crude ash, and crude fat; and there was a positive correlation among crude protein, crude ash, and crude fat.

### 2.7. Principal Component Analysis (PCA) Concerning Nitrogen Fixation, Nutritive Value, and Yield of Alfalfa

**Figure 6:**
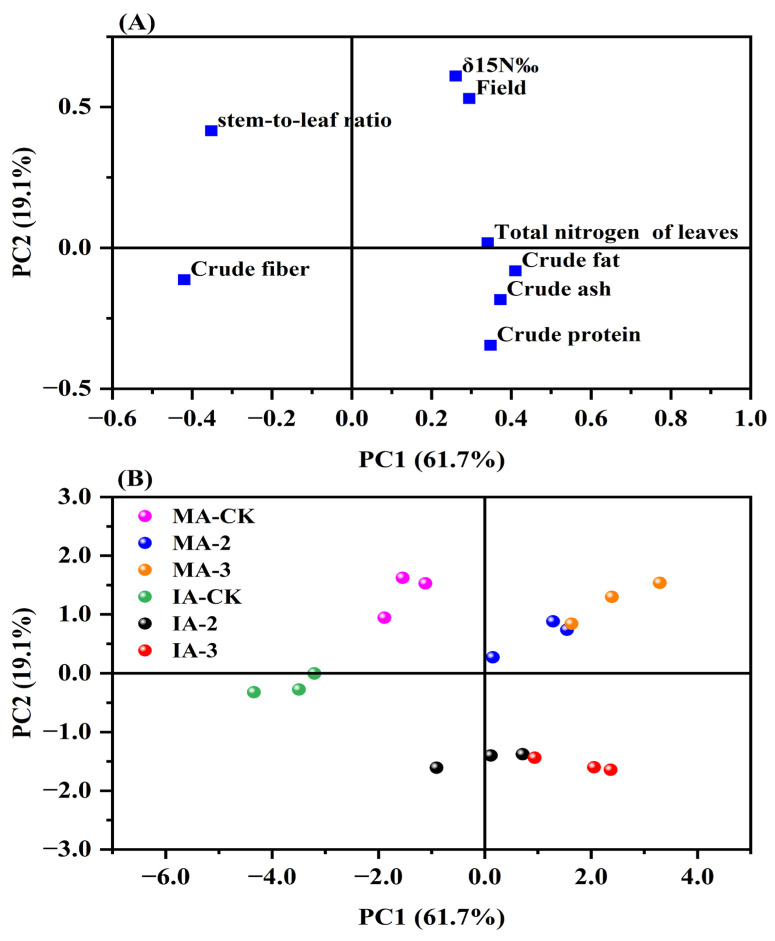
PCA loadings for 8 indexes related to forage quality (**A**), and scores for samples (**B**) under different mowing frequency treatments. MA-CK, MA-2, and MA-3 represent monocropped alfalfa subjected to mowing once, twice, and thrice, respectively; IA-CK, IA-2, and IA-3 represent intercropped alfalfa subjected to mowing once, twice and thrice, respectively.

The results of the principal component analysis (PCA) showed that the eight indexes, including the yield and nutritive values, could be concentrated into two principal components. The first and second principal components explained 61.7 and 19.1% of the variance in the dataset, respectively, with a cumulative variance contribution of 80.8%, indicating that these two principal components could provide comprehensive information on alfalfa yield and quality. Only the stem-to-leaf ratio and crude fiber were negatively correlated with PC1, and the rest of the indexes were positively correlated with PC1; crude protein, crude ash, crude fat, and crude fiber were negatively correlated with PC2, and the stem-to-leaf ratio, δ^15^N, yield, and total nitrogen of the leaves were positively correlated with PC2 (Figure 6A). Compared with the MA-CK treatment, both the MA-2 and MA-3 treatments significantly increased the PC1 score but had little effect on the PC2 score; compared with the IA-CK treatment, the IA-2 and IA-3 treatments significantly increased the PC1 score and decreased the PC2 score (Figure 6B).

## 3. Discussion

### 3.1. Root Distribution of Alfalfa

Vertical structural segregation and the ecological niche partitioning of the root system are recognized as crucial mechanisms for maintaining species coexistence and as essential prerequisites for fully leveraging the complementarity of intercropping systems [26]. Trees in intercropping systems generally have a longer lifespan than crops and tend to extend their roots deeper into the soil, while crop root systems often adopt a shallower distribution strategy to minimize overlap with tree root systems [27]. This trend is also evident in this study, where the center of gravity of the surface root distribution of intercropped alfalfa exhibited a significant upward shift compared to that of monocropped alfalfa (Table 1). Additionally, morphological indicators such as the root length density and root surface area of intercropped alfalfa were significantly lower than those of monocropped alfalfa, highlighting the negative impact of intercropping on the root system of alfalfa. These findings are consistent with those reported by Hadir et al. [28].

In this study, increased mowing frequency generally inhibited root development in alfalfa, possibly due to the depletion of root nutrients caused by the regeneration of aboveground biomass. This finding aligns with the results of a previous study by Mori et al. [29], which investigated the effects of mowing on early successional grasses and root systems. Additionally, Hakl et al. [30] compared the root morphological characteristics of alfalfa under management practices involving three and four cuts per year, revealing that four-cut management reduced root diameter and branching number. Interestingly, although mowing generally inhibited root development, it had a positive effect on the root system of alfalfa in the surface soil. To support plant growth and development, the root system needs to absorb a variety of essential mineral elements from the soil; therefore, plants must dynamically adjust their root structure to optimize nutrient acquisition [31].

### 3.2. Effect of Intercropping and Mowing on Biological Nitrogen Fixation

The determination of δ^15^N values using isotope mass spectrometry enables the quantification of subtle differences in δ^15^N abundance between non-nitrogen-fixing plants that thrive solely in mineral nitrogen environments and legumes that biologically fix nitrogen from the atmosphere [32]. In this study, the δ^15^N values of alfalfa in both the monocropping and intercropping systems were significantly lower than those of the non-nitrogen-fixing plant *Festuca elata*. Moreover, the δ^15^N values of alfalfa fell within the typical ranges reported in previous studies [33], which strongly attests to the effectiveness of alfalfa’s biological nitrogen-fixing capacity in this experimental area. We did not observe root nodule production during the 2021–2022 years of the trial; however, effective nodulation was detected in the third year (Supplementary Table S2). This delay may be attributed to the previous absence of legumes in the crop rotation on this experimental land, which resulted in fewer naturally occurring rhizobacteria in the soil, thereby hindering the symbiotic association with alfalfa roots. While the biological nitrogen-fixing capacity of legumes has been widely demonstrated to contribute nitrogen to crop–legume intercropping systems [34], research on nitrogen fixation in tree–legume intercropping systems remains limited. In this study, intercropping with walnut enhanced the biological nitrogen-fixing capacity of alfalfa, consistent with the findings of Querné et al. [17], thus confirming the feasibility of the tree–legume intercropping model.

This phenomenon may be related to the uptake of soil nitrogen by both walnut and alfalfa. Conventional wisdom suggests a negative correlation between soil nitrogen availability and biological nitrogen fixation by legumes [35], indicating that legumes typically prioritize the utilization of mineral nitrogen over atmospheric nitrogen [36]. In the walnut–alfalfa intercropping system, walnut growth and development consumed a substantial amount of mineral nitrogen, resulting in competition for nitrogen with the alfalfa. This scenario decreased soil nitrogen levels, which, in turn, may have enhanced the biological nitrogen fixation activity of alfalfa. Furthermore, this study found that a mowing-thrice treatment reduced the biological nitrogen-fixing capacity of alfalfa compared to CK and mowing-twice treatments. This reduction may be due to an excessive mowing frequency leading to a decline in root nutrients and a decrease in the total number of lateral roots containing alfalfa root nodules, subsequently resulting in fewer root nodules and effective root nodules [37]. This is also reflected in the variation in root morphology of alfalfa (Table 1). Sun’s study on soybean–grape intercropping systems indicated that mowing reduced biological nitrogen fixation in clover within a wheat–clover intercropping system [38]. Additionally, mowing increased the total nitrogen content of alfalfa leaves, suggesting a potential negative relationship between total leaf nitrogen content and biological nitrogen fixation.

### 3.3. Changes in Yield and Nutritive Value on Alfalfa

Many previous studies [39,40] have reported the yield advantage of intercropping (intercropping increases crop yield compared to monocropping), but our study found that intercropping resulted in a decrease in the yield of both walnut and alfalfa compared to the monocropping system. The reasons for these differences may be related to soil conditions and plant interactions. Viaud et al. [41] conducted a meta-analysis of sugarcane–legume intercropping and showed that intercropping with legumes resulted in yield changes in sugarcane ranging from −65% to +47%; Yang et al. [42] showed that both aboveground and belowground plant interactions affect yield in intercropping systems, with aboveground interactions such as shading having a more pronounced effect on yield. In this study, the monocropped walnut and monocropped alfalfa systems had higher soil nutrient background values and no species competition (Supplementary Table S1), which was more favorable for yield accumulation. Although intercropping reduced the respective yields of walnut and alfalfa, it significantly improved land use efficiency, with a LER calculated to be greater than 1 for all mowing frequency treatments, which fully demonstrated the excellent potential of intercropping to harmonize resource use and increase total system output. The positive effect of intercropping on land use efficiency was also found in the studies of Kimura et al. and Mason et al. [6,43]. An increase in mowing frequency increased alfalfa yield, which may be related to changes in root morphology that contribute to compensatory growth. Compensatory growth is a common response of plants to disturbances such as mechanical damage and has been demonstrated in a variety of plant studies [44]. Li et al. [45] argued that compensatory growth has been clearly demonstrated in studies on aspects of forest productivity, and Staalduinen et al. [46] showed that the stronger the potential for compensatory growth, the greater the persistence of the forage under grazing conditions. This is a good proof that in this study, the increase in mowing frequency led to changes in alfalfa root morphology (shallow root distribution), which prompted more nutrient transfer to the aboveground part, and therefore compensatory growth was accomplished. In addition, this study also found no significant difference in the LER among different mowing treatments in the intercropping system, indicating that mowing frequency had no effect on land use efficiency.

Nutritive value and yield are two important components in evaluating forage production, which is important for the development of livestock farming [47]. In this study, the increase in mowing frequency, besides having a positive effect on yield, also increased the nutritive value of alfalfa (increase in crude protein, crude ash, and crude fat content, and decrease in crude fiber content), which is similar to the findings of Li et al. and Čop et al. [48,49]. There may be two reasons for this: firstly, it is now widely recognized that extended forage growth time promotes biomass accumulation at the expense of stem and leaf nutrient concentrations [50], and increased mowing frequency reduced the time spent growing alfalfa each time compared to harvesting at the end of the growing season (CK), resulting in stem and leaf nutrient concentrations being maintained at a higher level; and secondly, the key driver of the nutrient value of stems and leaves is the stem-to-leaf ratio [51], and increased mowing frequency resulted in a significant decrease in the alfalfa stem-to-leaf ratio, which contributed to the accumulation of stem and leaf nutrients, i.e., stem-to-leaf ratio and nutritive value were highly significantly negatively correlated (Figure 6). The crude protein and other contents of the intercropped alfalfa were close to those of the monocropped alfalfa, which suggests that intercropping does not reduce the nutritive value of alfalfa, although it limits its yield.

Correlation analysis revealed a negative correlation between alfalfa yield and nitrogen fixation capacity (Figure 5). As biomass continues to increase, alfalfa requires more nitrogen, and a decrease in nitrogen-fixing capacity causes alfalfa to increase the proportion of mineral nitrogen uptake. In this experiment, no significant competition between walnut and alfalfa was observed across the three mowing treatments. We recommend increasing the mowing frequency in future studies to better investigate the interspecific relationships within the intercropping system.

## 4. Materials and Methods

### 4.1. Study Area

The study site was located in a 7-year-old walnut orchard in Jiyuan City, Henan Province, China, covering an area of about 150 hm^2^ that has a continental monsoon climate, with an average annual temperature and rainfall of 15.11 °C and 586.85 mm, respectively (meteorological data from Henan Xiaolangdi Forest Ecosystem National Observation and Research Station, China). The soil depth is 60–80 cm, the gravel content is 10–18%, the soil type is “Chromic Luvisol” [52], and the background values of the soil’s physicochemical properties are shown in Supplementary Table S1. The walnut trees in the orchard have a plant spacing (between trees) of 3 m and a row spacing of 8 m. Every year, the farmers carry out uniform pruning and care for the walnuts, and the walnut trees are basically the same in terms of growth, with an average diameter at breast height of 10.03 cm. The walnut trees were grafted, using black walnut (*Juglans nigra* L.) as the rootstock.

### 4.2. Experimental Design

Three types of sample plots were established in the walnut orchard to represent different systems: a monocropping walnut system (where only walnuts were planted and weeds were removed from the rows during the growth period), a walnut–alfalfa (*Medicago sativa* L. cv. Vernal) intercropping system (with alfalfa planted between the walnut rows), and a monocropping alfalfa system (where alfalfa was planted in open space approximately 1 km from the intercropping sample plots). Each system was replicated across three blocks. The experiment was conducted from March 2021 to October 2023, with the alfalfa planted in March 2021 using strip seeding at a row spacing of 50 cm, a seeding depth of 2 cm, and a seeding rate of 15 kg/hm^2^. Three treatments were implemented in both the monocropping alfalfa system and the intercropping system: mowing once (CK), mowing twice, and mowing thrice throughout the growing season. Each treatment was designated as MA-CK, MA-2, and MA-3 for the monocropping system, and IA-CK, IA-2, and IA-3 for the intercropping system. The walnut treatments in the intercropping system were identified as IW-CK, IW-2, and IW-3. Each treatment within a single plot consisted of three randomly distributed test zones (30 m × 16 m), resulting in a total of nine test zones for each mowing treatment. Isolation zones were established between the different test zones, and the specific layout of the plots and test zones is illustrated in Supplementary Figure S1. The CK treatment was harvested only at the end of September each year, the two-mowing treatment involved mowing at the beginning of flowering and harvesting at the end of September, and the three-mowing treatment added one additional mowing compared to the two-mowing treatment. The specific mowing schedule is presented in Supplementary Figure S2. To minimize disturbances, no fertilizers were applied during the 2021–2023 trial period, and the walnut trees were allowed to grow naturally without pruning. Rainfall was solely utilized to replenish soil moisture in the trial plots, with no irrigation measures taken, and weeds were managed manually. Sample collection of the aboveground parts of alfalfa was conducted on the day of each mowing to assess various indicators. To investigate the nitrogen fixation capacity of alfalfa, *Festuca elata* Keng ex E. B. Alexeev was selected as a non-nitrogen-fixing control plant. A separate sample plot (WF plot) was established in the orchard where *Festuca elata* was planted between the walnut rows concurrently with alfalfa, using a sowing rate of 30 kg/hm^2^. The WF sample plots were solely used for δ^15^N value measurements, and due to the absence of alfalfa nodules in 2021 and 2022, simultaneous collections of alfalfa and *Festuca elata* were conducted only in 2023.

### 4.3. Measurements and Calculations

#### 4.3.1. Meteorological Data Monitoring

An automatic microclimate observation system was installed in the orchard to provide continuous observation of air temperature, relative humidity, and rainfall. HMP45C (Campbell Inc., Northwood, OH, USA), A100LM (Vector Inc., London, UK), and TE525M (Texas Inc., Dallas, TX, USA) sensors were used, and the datalogger used was the CR10X system (Campbell Inc., Northwood, OH, USA). The data were collected every 1 min, and the average value for one group was output every 10 min. The temperature and rainfall data for 2021–2023 are shown in Supplementary Figure S3.

#### 4.3.2. Root Collection and Determination

Alfalfa root was collected at the end of the 2023 growing season in the different mowing treatments (three randomly selected test zones per treatment) in the monocropping alfalfa and intercropping systems using a root sampler (5 cm in diameter, 20 cm in depth, and 392.5 cm^3^ in volume) for root samples from four soil layers (0–20, 20–40, 40–60, and 60–80 cm). After cleaning the alfalfa root samples, they were scanned using a root scanner (V850, EPSON, Nagano, Japan), and a WinRHIZO (Regent Instruments Inc., Quebec, QC, Canada) root analysis system was used to determine the root length, root surface area, root average diameter, and root volume data for each layer of alfalfa. The root length density data of alfalfa are the ratio of the total root length to volume (392.5 cm^3^). The root distribution data of alfalfa in each soil layer is the average of the root data for each soil layer in the three test zones.

#### 4.3.3. Sample Collection and δ^15^N Determination

Plant samples were collected in May, July, and September of 2023 as follows: ten alfalfa plants were randomly selected from each mowing treatment in all monocropped and intercropped alfalfa sample plots, resulting in a total of 30 alfalfa plants per treatment; 30 aboveground portions of *Festuca elata* were randomly collected from the WF sample plots. The collected aboveground portions of both alfalfa and *Festuca elata* were dried and pulverized to obtain stem and leaf samples. The δ^15^N values of these samples from each treatment were determined using a stable isotope ratio mass spectrometer. The δ^15^N value for alfalfa across the different mowing treatments represented the average of the δ^15^N values from the 30 sampled plants, while the δ^15^N value for *Festuca elata* was the average of the δ^15^N values from the 30 sampled *Festuca elata* plants. ^15^N is a stable isotope of the element nitrogen with a slightly heavier mass relative to the common ^14^N. In mass spectrometry analysis, the ionic mass of nitrogen ^15^N is clearly distinguishable from that of nitrogen ^14^N, which provides scientists with the tools to accurately distinguish and measure the two [53]. ^15^N is widely used in nitrogen fixation studies in plants [54]. Using the ^15^N natural abundance method, the proportion of nitrogen acquired from the atmosphere by nitrogen-fixing plants can be calculated using the following equation [55]:

$$\%Ndfa=(\delta^{15}Ns-\delta^{15}Nf)/\left(\delta^{15}Ns-\delta^{15}Na\right),$$

where δ^15^Ns represents the abundance of ^15^N in the reference plant (*Festuca elata* under the same treatment), δ^15^Nf represents the abundance of ^15^N in the alfalfa, and δ^15^Na represents the abundance of ^15^N in the nitrogen-free sand-culture alfalfa.

We were unable to accurately calculate the percentage of nitrogen fixation due to a lack of alfalfa δ^15^Na values suitable for our experimental conditions and varieties, but this Equation suggests that alfalfa’s δ^15^N value and the percentage of nitrogen fixation are negatively correlated, and therefore the determination of alfalfa’s δ^15^N value can characterize the nitrogen-fixing capacity of alfalfa. This was also demonstrated in the study by Querné et al. [17] where the δ^15^N approach allowed valuable biological nitrogen fixation activity estimations when used to compare different cropping situations. Given that the thrice-mowing treatment was not completed until the final harvest of alfalfa, in order to compare the effect of mowing frequency on nitrogen fixation, we used our data on the δ^15^N values measured in September for analysis of variance.

#### 4.3.4. Stem-to-Leaf Ratio Calculation and Total Nitrogen Determination of Leaves

A total of three sampling events were conducted each year. During the first and second mowings, 15 plants were randomly selected for aboveground portion sampling from each monocropped and intercropped alfalfa plot, resulting in a total collection of 45 monocropped and 45 intercropped alfalfa plants. At the time of the final harvest, three test zones were randomly selected for sampling the aboveground portions from 45 alfalfa plants in each treatment of both the monocropping and intercropping systems. The collected aboveground portions were dried, and each plant was separated into stem and leaf components, which were weighed individually. The stem-to-leaf ratio for each plant was calculated as the ratio of the dry weight of the stem to the dry weight of the leaf. The stem-to-leaf ratio data obtained from each mowing represented the averages of the stem-to-leaf ratios from the 45 sampled alfalfa plants. The stem-to-leaf ratio for the CK treatment was recorded at the final harvest (the third mowing), while the ratio for the two-mowing treatment was the average of the ratios from the first mowing and the final harvest. The ratio for the three-mowing treatment was the mean of the ratios from all three mowing events. The data used for the statistical analysis of the stem-to-leaf ratios from each treatment were averaged over three years (2021–2023). The Kjeldahl nitrogen method [56] was employed to determine the total nitrogen content of the leaves after the leaf samples were dried and pulverized, and the total nitrogen content data for each treatment reflected the average from 2021 to 2023.

#### 4.3.5. Measurement of Nutritive Indexes

Samples were taken a total of three times per year: at the first and second time of mowing, an additional 15 alfalfa plants were randomly selected for aboveground portion sampling in each monocropped and intercropped alfalfa sample plot, and 45 plants were collected, respectively; at the final harvest, three test zones were randomly selected for aboveground portion sampling for each treatment of the monocropping and intercropping systems, and a total of 45 alfalfa plants were collected. The aboveground portions of the single plants were dried and crushed to obtain a mixed sample of single alfalfa stems and leaves for a determination of crude protein, crude ash, crude fat, and crude fiber content. Crude protein was determined by the Kjeldahl nitrogen determination method [56]; crude ash was determined by the high-temperature scorching method, which was referred to by Bao [57]; crude fat was determined by the Soxhlet extraction method, which was referred to by Bao [57]; and crude fiber was measured by the filtration method (GB/T 5009.10-2003, China). The crushed sample was transferred into a conical flask, then 200 mL of boiled 1.25% H_2_SO_4_ solution was added, and the first filtration was carried out. Next, the filtered residue was mixed with 200 mL of a boiled 1.25% KOH solution, and the remaining residue was dried and weighed to determine the crude fiber content after the second filtration [55].

Nutrient index data statistics for each treatment were consistent with the descriptions in the previous subsection, and the crude protein, crude ash, crude fat, and crude fiber contents were averaged from the 2021–2023 data.

#### 4.3.6. Calculation of Yield and Land-Equivalent Ratios

During the first and second mowings of each year, 20 sample areas (1 m^2^) were randomly established to collect the aboveground portions of alfalfa. These samples were dried and weighed to obtain the biomass per square meter for both the monocropped and intercropped alfalfa. The average biomass from the 20 samples was then multiplied by the area to estimate the yield per mowing (per hectare). At the time of the final harvest, three test zones were randomly selected for each treatment of the monocropped and intercropped alfalfa plots, and 15 sample areas (1 m^2^) were established to measure the biomass, following the same method as described previously to estimate the yield of each treatment at the final harvest. The total annual yield for the alfalfa in the CK treatment corresponded to the yield at the final harvest. For the mowing-twice treatment, the yield was the sum of the yields from the first mowing and final harvest, while for the mowing-thrice treatment, it was the sum of the yields from the first and second mowings as well as the final harvest. Walnuts were harvested in late August each year, and 10 walnut trees were randomly selected from each treatment in the monocropped and intercropped walnut plots. All fruits were collected, de-greened, dried, and weighed to calculate the average dry weight of the fruits per single walnut tree. This figure was then multiplied by the planting density of the walnuts (416.67) to estimate the annual production of walnuts. The land equivalent ratio data were averaged over the years 2021–2023 and calculated using the following formula [58]:

$$LER={(YW}_{intercropping}/{YW}_{monocropping})+\left({YA}_{intercropping}/{YA}_{monocropping}\right),$$

where YW_intercropping_ represents the yield of the walnut in the intercropping system, YW_monocropping_ represents the yield of the walnut in the monocropping system, YA_intercropping_ represents the yield of the alfalfa in the intercropping system, and YA_monocropping_ represents the yield of the alfalfa in the monocropping system.

#### 4.3.7. Data Analysis

SPSS22.0 software was used to complete the normality (Shapiro–Wilk test) and chi-square (Levene’s test) tests on the raw data; Fisher’s Least Significant Difference test and Duncan’s Multiple Comparison Analysis were used to complete the significance analyses of the indexes under different mowing frequencies. The correlation analysis and principal component (PCA) analysis were completed using Origin2021, and all images were created by Origin2021.

## 5. Conclusions

Intercropping and mowing frequency influenced the nitrogen fixation, nutritive value, and yield of alfalfa. Intercropping with walnut enhanced the biological nitrogen fixation capacity of alfalfa without diminishing its nutritive value. Increased mowing frequency stimulated surface root development and positively affected alfalfa’s total nitrogen content in its leaves, its nutritive value, and its yield, despite generally inhibiting alfalfa’s root distribution. However, the mowing-thrice treatment limited alfalfa’s biological nitrogen fixation capacity, potentially impacting mineral nitrogen uptake in the soil. Additionally, although intercropping resulted in lower yields for both walnut and alfalfa, the land equivalent ratios for all three mowing treatments exceeded 1.0, indicating that intercropping can enhance land use efficiency, and this effect was not influenced by mowing frequency. Therefore, intercropping walnut and alfalfa is a viable practice in this region and can be optimized through three mowings to achieve the best system yield and nutrient value.

We did not observe a competitive relationship between walnut and alfalfa under the current mowing treatment setup. To better understand the interspecific nitrogen relationships within the intercropping system, we recommend increasing the mowing frequency in subsequent experiments and evaluating its impact on yield and nutritive value. The results of this study can serve as a reference for agroforestry management in similar areas.

## Figures and Tables

**Table 1:** Root morphological characteristics of alfalfa at different soil depths.

Soil Depth cm		Root Length Density	Root Surface Area/cm^2^	Root Diameter/mm	Root Volume/cm^3^
0–20	MA-CK	1.15 ± 0.11 b	171.85 ± 9.20 b	3.19 ± 0.11 a	9.99 ± 0.15 b
	MA-2	1.35 ± 0.13 a	198.20 ± 9.25 a	3.21 ± 0.14 a	10.37 ± 0.10 a
	MA-3	1.37 ± 0.14 a	205.46 ± 10.61 a	3.16 ± 0.15 a	10.44 ± 0.13 a
	IA-CK	0.83 ± 0.09 c	147.15 ± 7.86 c	2.12 ± 0.13 b	8.55 ± 0.18 d
	IA-2	1.06 ± 0.08 b	168.54 ± 9.12 b	2.08 ± 0.16 b	8.92 ± 0.24 c
	IA-3	1.04 ± 0.08 b	164.79 ± 7.22 b	2.09 ± 0.14 b	8.89 ± 0.23 c
	Mean	1.13 ± 0.21	176.00 ± 21.88	2.64 ± 0.57	9.53 ± 0.80
20–40	MA-CK	0.88 ± 0.12 a	147.28 ± 9.20 a	2.21 ± 0.09 a	7.88 ± 0.11 a
	MA-2	0.92 ± 0.14 a	142.70 ± 9.52 ab	1.82 ± 0.11 b	7.82 ± 0.12 a
	MA-3	0.85 ± 0.14 ab	147.17 ± 7.33 a	1.54 ± 0.15 c	7.82 ± 0.09 a
	IA-CK	0.65 ± 0.08 b	123.23 ± 6.74 c	1.72 ± 0.11 bc	6.45 ± 0.13 b
	IA-2	0.67 ± 0.08 b	125.12 ± 9.47 c	1.53 ± 0.09 c	6.39 ± 0.10 b
	IA-3	0.67 ± 0.06 b	127.71 ± 9.67 bc	1.32 ± 0.12 d	6.34 ± 0.05 b
	Mean	0.77 ± 0.15	135.54 ± 12.95	1.69 ± 0.30	7.12 ± 0.75
40–60	MA-CK	0.58 ± 0.06 a	97.82 ± 13.78 a	1.68 ± 0.11 a	5.72 ± 0.15 a
	MA-2	0.47 ± 0.05 b	80.59 ± 10.02 b	1.23 ± 0.16 b	5.23 ± 0.11 b
	MA-3	0.36 ± 0.08 c	63.08 ± 2.54 c	0.85 ± 0.17 cd	4.81 ± 0.09 c
	IA-CK	0.47 ± 0.06 b	79.99 ± 11.07 b	1.26 ± 0.10 b	4.05 ± 0.08 d
	IA-2	0.31 ± 0.04 cd	63.12 ± 3.83 c	0.97 ± 0.14 c	3.57 ± 0.12 e
	IA-3	0.24 ± 0.04 d	45.41 ± 5.77 d	0.67 ± 0.09 d	3.15 ± 0.14 f
	Mean	0.41 ± 0.12	71.67 ± 18.70	1.11 ± 0.35	4.42 ± 0.94
60–80	MA-CK	0.33 ± 0.05 a	46.03 ± 6.24 a	1.06 ± 0.08 a	2.77 ± 0.15 a
	MA-2	0.20 ± 0.04 b	33.11 ± 4.34 b	0.76 ± 0.07 b	1.77 ± 0.18 b
	MA-3	0.12 ± 0.02 c	18.19 ± 5.94 cd	0.47 ± 0.08 c	0.86 ± 0.14 d
	IA-CK	0.20 ± 0.03 b	30.44 ± 7.92 b	0.73 ± 0.05 b	1.65 ± 0.12 b
	IA-2	0.10 ± 0.03 c	20.62 ± 2.88 c	0.48 ± 0.07 c	1.23 ± 0.15 c
	IA-3	0.04 ± 0.02 d	10.06 ± 2.69 d	0.30 ± 0.07 d	0.55 ± 0.14 e
	Mean	0.17 ± 0.10	26.41 ± 12.80	0.63 ± 0.26	1.47 ± 0.75

Different lowercase letters indicate significant differences (*p* < 0.05), and error bars represent standard deviation. MA-CK, MA-2, and MA-3 represent monocropped alfalfa subjected to mowing once, twice, and thrice, respectively; IA-CK, IA-2, and IA-3 represent intercropped alfalfa subjected to mowing once, twice, and thrice, respectively.

## Data Availability

The datasets generated and/or analyzed during the current study are available from the corresponding author on reasonable request.

## Supplementary Materials

The following supporting information can be downloaded at: https://www.mdpi.com/article/10.3390/plants14020240/s1, Figure S1: Schematic diagram of mowing treatment; Figure S2: Experimental layout diagram: (A) monocropping walnut system, (B) monocropping alfalfa system and (C) walnut-alfalfa intercropping system; Figure S3: Monthly variation of precipitation; Table S1: Background value of soil physical and chemical properties; Table S2: Root nodule numbers and δ^15^N values of *Medicago sativa* L. and *Festuca elata* in 2023.
