# Supplementary material for: Effects of Intercropping and Mowing Frequency on Biological Nitrogen Fixation Capacity, Nutritive Value, and Yield in Alfalfa (Medicago sativa L. cv. Vernal)
Source: Plants (Basel). 2025 Jan 16;14(2):240. doi: 10.3390/plants14020240 (PMC11768302; doi:10.3390/plants14020240)
Supplement: Supplementary file 1 [file plants-14-00240-s001.zip › plants-3387510-supplementary.pdf]

Supplementary Table S1 Root nodule numbers and $\delta^{15}N$ values of *Medicago sativa* L. and *Festuca elata* in 2023.

	Number of root nodules	$\delta^{15}N$ value/ ‰
MA-CK	13.33	-1.26 ±0.03
MA-2	11.67	-1.29 ±0.02
MA-3	6.00	-0.55 ±0.04
IA-CK	17.67	-2.30 ±0.08
IA-2	15.67	-2.19 ±0.03
IA-3	9.00	-1.82 ±0.10
Festuca elata	-	1.73 ± 0.20

Supplementary Table S2 Background value of soil physical and chemical properties.

	Soil depth cm	Organic Carbon %	pH	Total Nitrogen %	Total Phosphorus %	Total Potassium %	Effective phosphorus mg/kg	Effective potassium g/kg	Soil capacity g/cm^3
Walnut sample plot	0-20	1.07±0.06a	7.12±0.05b	0.11±0.01a	0.09±0.001a	2.27±0.22a	26.63±1.58a	0.23±0.05a	1.56±0.08a
	20-40	0.75±0.07b	7.28±0.04ab	0.08±0.01b	0.07±0.001b	2.24±0.14a	11.28±0.73b	0.14±0.03a	1.55±0.07a
	40-60	0.57±0.08c	7.51±0.05ab	0.07±0.001bc	0.07±0.001bc	2.04±0.04a	6.82±0.34b	0.13±0.02a	1.60±0.22a
	60-80	0.44±0.04c	7.73±0.03a	0.05±0.001c	0.07±0.001c	2.02±0.08a	5.53±0.28b	0.12±0.01a	1.73±0.17a
Alfalfa sample plot	0-20	1.12±0.03a	6.97±0.35a	0.13±0.001a	0.08±0.001a	2.53±0.16a	19.42±1.25a	0.30±0.00a	1.51±0.20a
	20-40	0.75±0.02b	7.00±0.18a	0.10±0.001b	0.06±0.001b	2.52±0.10a	11.55±0.53b	0.13±0.01b	1.66±0.14a
	40-60	0.68±0.08b	7.23±0.27a	0.08±0.001c	0.05±0.001c	2.50±0.10a	4.55±0.50c	0.12±0.00c	1.66±0.07a
	60-80	0.33±0.01c	7.45±0.20a	0.05±0.001d	0.05±0.001c	2.37±0.10a	3.40±0.28c	0.10±0.00d	1.68±0.04a

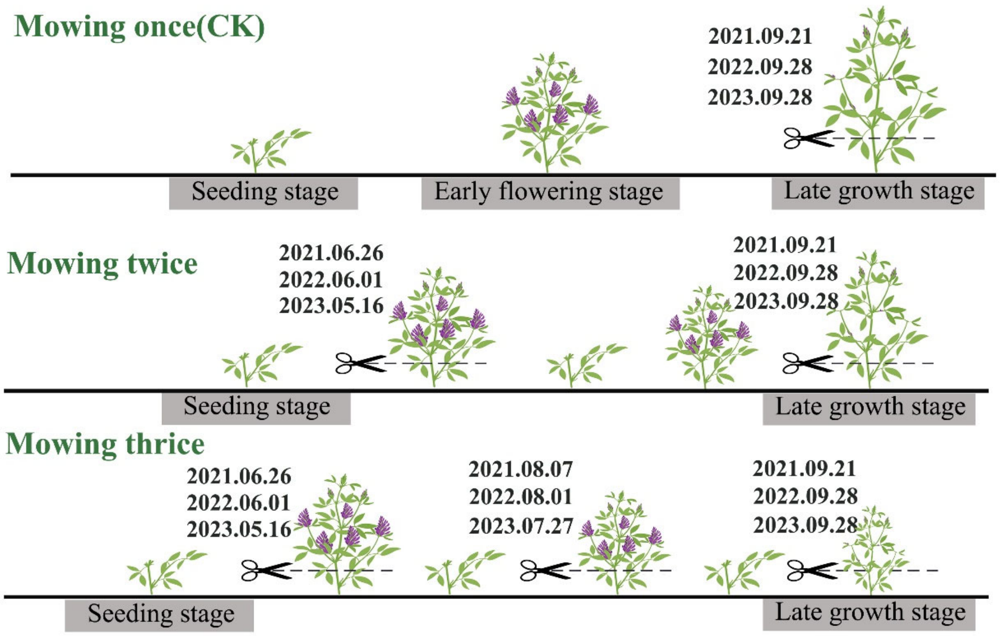

Supplementary Figure. S1 Schematic diagram of mowing treatment.

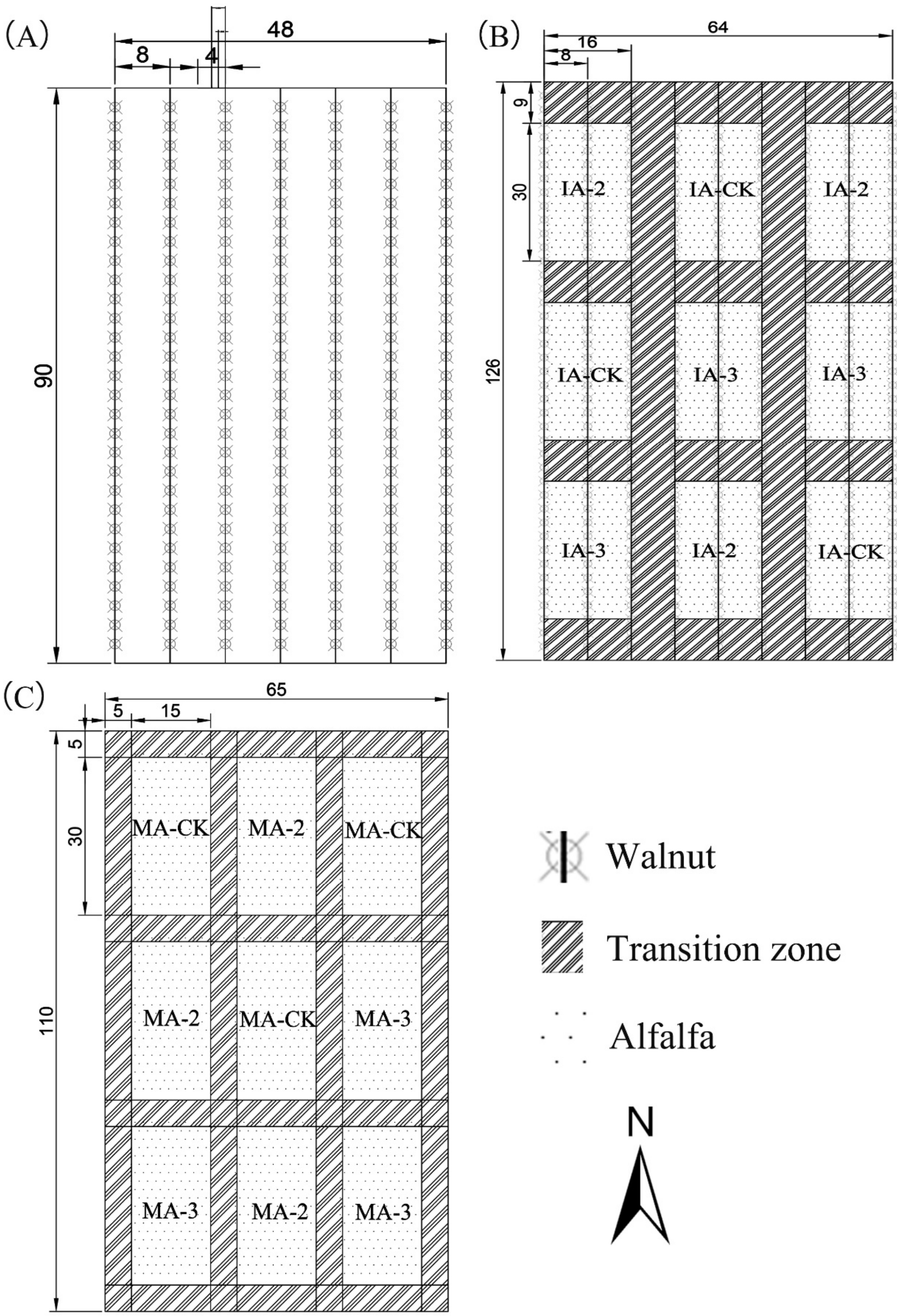

Supplementary Figure. S2 Experimental layout diagram: (A) monocropping walnut system, (B) monocropping alfalfa system and (C) walnut-alfalfa intercropping system.

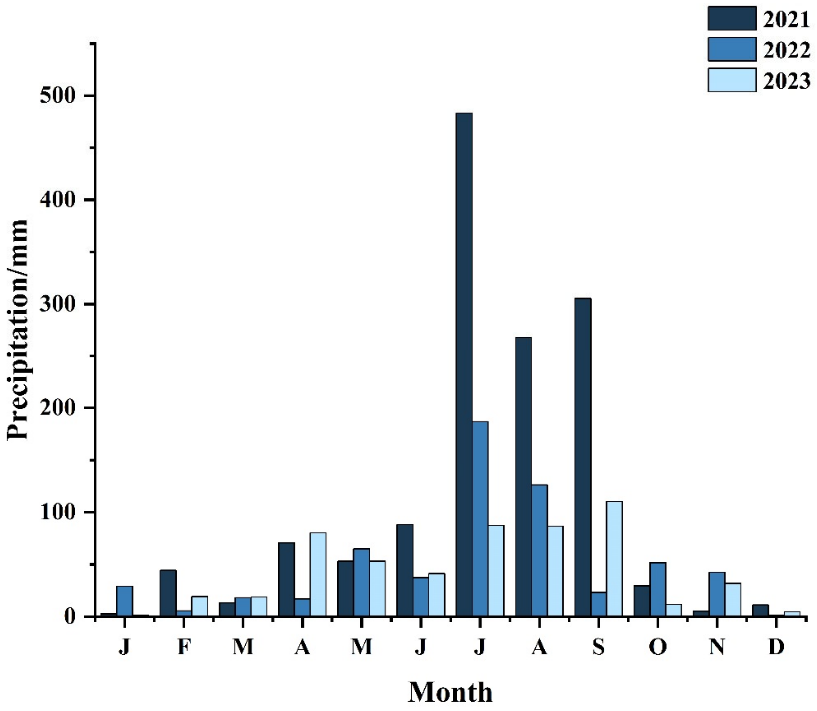

Supplementary Figure. S3 Monthly variation of precipitation.
